# Supplementary material for: Orchid species diversity across a forest disturbance gradient in west Mau forest, Kenya
Source: PLoS One. 2024 Aug 7;19(8):e0307887. doi: 10.1371/journal.pone.0307887 (PMC11305582; doi:10.1371/journal.pone.0307887)
Supplement: Attachment — Submitted filename: Response to Review Comments.doc [file pone.0307887.s002.doc]

|  | **REVIEW COMMENTS TO THE AUTHOR** | **AUTHOR RESPONSE** |
| --- | --- | --- |
| **1** | The methods section does not explain well the study design nor the performed analysis | We have improved the section to address the concerns raised. New text is highlighted in yellow in the method section. |
| **2** | The main setup of the study is comparing orchid diversity along a degradation gradient.  However:   a) no information is provided how the 3 sites representing this gradient were selected, | The concern has been addressed by expounding on the set up of the study in the study design section – lines 85 to 96. The revised section has been highlighted in yellow. |
|  | b) how far apart they are, | This has been addressed in the study design section - lines 92 to 94. The revised section has been highlighted in yellow |
|  | c) to what extent they differ in degradation level (how do they e.g. defer in forest structure?), | This has been addressed in the study design section - lines 86 to 91. The revised section has been highlighted in yellow |
|  | d) if they are similar otherwise (e.g. same soil type, topography, etc.), | The topography, geology and soil are similar – lines 76 to 78 in study area section. The revised section has been highlighted in yellow |
| **3** | Of what I do understand from the description of the scientific setup, only 1 site per degradation level is chosen if I understand correctly. This seems to me not enough. | Three sub-blocks were selected from each of the forest disturbance regimes – line 97-98. The revised section has been highlighted in yellow.  In order to avoid the edge effect caused by narrowness of some sub-blocks (100 m to 150 m wide), only one transect was laid in each of the three sub-blocks under a forest disturbance regime – lines 99-101. The revised section has been highlighted in yellow. |
| **4** | Regarding the analysis itself, the description is also very short. It is for example not clear to me how orchid density per tree type was calculated. Why is the unit per ha and not per tree? | The concern has been addressed by expounding on the description of data analysis – lines 125 to 131. Although data were collected on trees at the plot level, degradation occurred at the landscape level, hence the presentation of orchid results has been done at ha level. The revised section has been highlighted in yellow. |
| **5** | Another major concern that I have is that many statements are made in the discussion that are not backed up by data nor references (e.g. regarding the importance of rough bark for orchids). I recommend reformulating to make clear it concerns speculation, and/or back statements up by data that is presented in the results section. | We have addressed this concern by introducing results of symbiotic relationships and bark texture of orchid host trees – lines 199 to 206. We have also included table 4: Bark texture of orchid host trees and respective orchid clumps. The revised section has been highlighted in yellow. |
| **6** | In the study design section: It is not clear how many line transects were established per site nor how many subplots per plot nor how the number of 45 sample plots was reached. | This has been expounded on in lines 97 to 108 and the revised section has been highlighted in yellow. |
| **7** | In the data collection section: It is not clear to me at which level the data was collected. Was this per host tree? | Data were collected on trees within a sample plot. The data were converted to ha level because forest degradation took place at landscape level. |
| **8** | In the data analysis section: It is not clear to me at which level species diversity and abundance was calculated, was this at the tree or plot level? Further, were any covariates taken into account in the statistical analysis? Any dependence structures tested? | The standard practice is to convert data from plot level to ha level because analysis is about obtaining means.  A detailed description has been provided in lines 125 to 131 and revised section has been highlighted in yellow |
| **9** | The conclusion reads more as a recommendation than a conclusion. | The conclusion has been rewritten – lines 285 to 291. The revised section has been highlighted in yellow |
