# Supplementary material for: Orchid species diversity across a forest disturbance gradient in west Mau forest, Kenya
Source: PLoS One. 2024 Aug 7;19(8):e0307887. doi: 10.1371/journal.pone.0307887 (PMC11305582; doi:10.1371/journal.pone.0307887)
Supplement: Attachment — Submitted filename: A rebuttal letter.doc [file pone.0307887.s003.doc]

|  | **Review Comments to the Author** | **Author Response** |
| --- | --- | --- |
| 1 | Table including a classification of the three different forest disturbance regimes | This table has been provided, designated as table 1 and highlighted in yellow in the document (Line 91-92, 103-106) |
| 2 | Sampling and identification of orchids in more detail and a photo plate of the orchid species given. | More details of sampling method have been added and highlighted in yellow. An orchid photo has also been included (Line 123-125, 130-137) |
| 3 | A map of the study area | A map of the study site has been included (Line 72, 84-86) |
| 4 | Form (epiphytic or terrestrial) of the orchids and also the family and bark type of each host tree. | A column on the host tree family has been introduced in table 5 and the orchids described here are epiphytes (Line 27-239, 298-299) |
| 5 | Table of data on the height of each clump on the host tree or within vertical zones | Data on the height of each clump on the host tree in Table 6 and described (Line 230-235, 241-242, 305-308) |
| 6 | Concluding statements should be supported by references | More citations/references have been included to support the conclusions (Line 298, 306-308, 316 and 319) |

**AUTHOR RESPONSE**
